# XIST and TSIX: Novel Cancer Immune Biomarkers in PD-L1-Overexpressing Breast Cancer Patients

**DOI:** 10.3389/fonc.2019.01459

**Published:** 2020-01-10

**Authors:** Esraa A. Salama, Reda E. Adbeltawab, Hend M. El Tayebi

**Affiliations:** ^1^Molecular Pharmacology Research Group, Department of Pharmacology and Toxicology, Faculty of Pharmacy and Biotechnology, German University in Cairo, Cairo, Egypt; ^2^Department of Surgery, Faculty of Medicine, Ain Shams University, Cairo, Egypt

**Keywords:** non-invasive immune biomarkers, XIST, TSIX, PD-L1, liquid biopsies, lncRNAs

## Abstract

Escaping antitumor immunity is a hallmark in cancer progression. Programmed cell death protein 1 (PD-1) is an immune checkpoint receptor responsible for the maintenance of immune tolerance; PD-1 ligand (PD-L1) is overexpressed in tumor cells, simplifying their escape from the immune system through T-cell function suppression. Notwithstanding that cancer antigen (CA)125, carcinoembryonic antigen (CEA), CA15-3, and alpha-fetoprotein (AFP) are among conventional breast cancer diagnostic biomarkers, their lack of sensitivity and specificity resides among their major limitations. Furthermore, human epidermal growth factor receptor (HER)2 and interleukin (IL)-6—demonstrated as breast cancer immune biomarkers—still possess limitations, for instance, technical detection problems and stability problems, which necessitate the discovery of novel, stable non-invasive cancer immune biomarkers. XIST and TSIX are two long non-coding (lnc)RNAs possessing a role in X chromosome inactivation (XCI) as well as in breast cancer (BC). In the present study, they were investigated as stable non-invasive breast cancer immune biomarkers. The study demonstrated that PD-L1 was overexpressed in the different molecular subtypes of breast cancer patients as well as in MDA-MB-231 cells. Furthermore, lncRNAs XIST and TSIX were markedly increased in the tissues, lymph nodes, and different body fluids of breast cancer patients compared to controls. In addition, XIST and TSIX were differentially expressed in subtypes of BC patients, and their levels were correlated to PD-L1 expression level. In conclusion, this correlative study has shed light on the role of both lncRNAs XIST and TSIX as potential non-invasive BC immune biomarkers reflecting the evaded immune system of the patient and overcoming the instability problem of common BC biomarkers.

## Introduction

Breast cancer (BC) is the most commonly diagnosed cancer in females and has the highest fatality rate among other cancer types (1), competing with heart disease in being the leading cause of death worldwide (2). BC is classified into five subtypes: luminal A, luminal B, luminal B-like, human epidermal growth factor receptor (HER)2, and triple negative (3). The triple-negative BC (TNBC) subtype is the most aggressive subtype, and it is further classified into four subtypes: luminal/androgen receptor (LAR), mesenchymal (MES), basal-like immune suppressed (BLIS), and basal-like immune activated (BLIA) subtypes (4). The triple-negative subtype was demonstrated in several studies to frequently express the programmed cell death-ligand 1 (PD-L1) (5, 6); there exist an overall immune system dysfunction and suppression throughout different BC subtypes due to the abundance of immune suppressive cells, namely, regulatory T cells (Tregs), and myeloid-derived suppressor cells (MDSCs), within the BC tumor microenvironment (7).

PD-L1 and its receptor programmed cell death protein 1 (PD-1) are immune checkpoint regulators promoting self-tolerance by protecting the body from the excessive T-cell activity, inflammation, and autoimmunity (8). This is maintained through PD-1 (expressed on T cells)/PD-L1 (expressed mainly in nonlymphoid organs) ligand binding leading to T-cell suppression (9). In malignant state, the PD-1/PD-L1 signaling pathway is utilized as an immune escape mechanism for the tumor cells through the overexpression of PD-L1 by the cancer cells causing T-cell suppression and immune system escape (10). PD-L1 involvement in BC was highlighted in several studies. A recent study has postulated that TNBC tumors evade the immune system through upregulation of PD-L1 on the surface of TNBC cancer stem cells *via* the activation of WNT pathway (11). Another study demonstrated that PD-L1 expression in BC has been associated with grade 3 (G3), triple-negative subtype, and worse prognosis (12). Furthermore, the upregulation of PD-L1 together with lactate dehydrogenase A (LDHA) in TNBC patients was related to poor patient outcome (5). Due to the involvement of the immune system in cancer progression, the emergence of immunotherapy represented a powerful weapon exclusively targeting dividing tumor cells as well as dispersed metastasis (13). The PD-L1 monoclonal antibody atezolizumab (14) and the eradication of TNBC through targeting glycosylated PD-L1 (15) signify successful examples of PD-L1 utilization in immunotherapy. Furthermore, cancer immune biomarker usage emerged from their ability to reflect patient immune status and his/her eligibility to immune therapy (16), in addition to the lack of sensitivity and specificity of conventional BC diagnostic biomarkers carcinoembryonic antigen (CEA), cancer antigen (CA)125, CA15-3, and alpha-fetoprotein (AFP) (17). Although, PD-L1 expression (18), high tumor mutation load (19), HER2 (20), and interleukin (IL)-6 (21) were successful illustrations of tumor and BC immune biomarkers, their technical detection problems (22) and instability in extended cryopreservation blood samples (23) have urged the discovery of novel stable immune biomarkers, the long non-coding (lnc)RNAs. To date, lncRNAs execute pivotal roles as cancer biomarkers as they are highly sensitive, specific, and stable in different body fluids, especially if they were circulating enclosed within apoptotic bodies or exosomes (24). It was reported in Shi et al. (25) that the lncRNAs successfully resisted ribonuclease enzyme as they were effectively detected in different body fluids where ribonuclease enzymes were present in rich quantities.

XIST and TSIX are two lncRNAs with a pivotal role in X chromosome inactivation (XCI) (26, 27) as well as in BC (28, 29). XCI is an important mechanism that compensates dosage disequilibrium introduced by the heteromorphic nature of the X and Y sex chromosomes in mammals so that only one X chromosome is transcriptionally active in both male and female cells. XCI is essential for the proper development and cellular differentiation as the presence of two active X chromosomes is correlated with a poorly differentiated state (30). In mice, XCI occurs in two waves. The initial wave commences in morula in preimplantation stage where lncRNA XIST expression is restricted only to inherited paternal silenced or imprinted (Xi) X chromosome. This is followed by lncRNA XIST expression suppression in all inner cell mass cells of blastocyst and Xi becomes reactivated. Finally, during embryo implantation, second wave of XCI initiates. This wave is characterized by random XCI where silencing of the X chromosome occurs through expression of lncRNA XIST in cis exclusively from future X inactive chromosome (Xi); this occurs through expression of lncRNA TSIX antisense to XIST, ensuring its repression from future active X chromosome (Xa) (31). In humans, XCI occurs similarly, yet with some discrepancies (31). Instead of TSIX gene, which has been marked in human genome, however, with no transcriptional evidence in human preimplant embryo, a different candidate lncRNA called X active coating transcript (XACT) was reported to be responsible for inhibition of XIST ability to silence X chromosome (31). To date, in humans, limited uncertain evidence for Xi status in cancer is accessible (32). It was reported in one study that in BC, multiple X chromosomes (XXX or XXXX) were observed (33). However, another recent study postulated that the inactive X chromosome is epigenetically labile, which results in a double dose of X chromosome genes favoring cancer development (32). As for the contribution of lncRNAs XIST and TSIX in BC, their precise role is largely unknown with minimal studies reporting lncRNA XIST as a tumor suppressor lncRNA whose level is downregulated in BC (28, 29), with no suggestions about lncRNA TSIX role in BC. In this study, we aimed to investigate the role of lncRNAs XIST and TSIX as stable non-invasive BC immune biomarkers in different body fluids of BC patients and correlate their expression to PD-L1 expression.

## Materials and Methods

### Sample Collection

BC biopsies, lymph nodes (LN), whole blood, serum, and nipple discharge (ND) were collected from 42 BC patients. It should be noted that nipple discharge collection was not available for all BC subtypes in this study. All BC patients were included based on the exclusion criteria (no other comorbidities other than BC). All samples were stored at −80°C until further use. All subjects gave their written informed consent, and the Ain Shams University ethical review committee approved the study. The study followed the ethical guidelines of the 1975 Declaration of Helsinki. Peripheral blood mononuclear cells (PBMCs) were isolated from whole blood using Ficoll density gradient technique. Patients' clinical parameters are presented in Table 1 and Supplementary Table 1.

**Table 1:** Patients characteristics.

**Patients (*n* = 42)**	**Percentage**
**Gender**	
Female 40/42	95%
Male 2/42	5%
**Age**	
Lower than 50 years old (15/42)	35.7%
More than 50 years old (27/42)	64.2%
**Family history**	
Positive family history (10/42)	23.8%
Negative family history (32/42)	76.19%
**ER**	
ER + VE (30/42)	71.42%
ER – VE (12/42)	28.57%
**PR**	
PR + VE 30/42	71.42%
PR – VE 12/42	28.57%
**HER2/NEU**	
HER2 + VE 8/42	19%
HER2 – VE 34/42	80%
**LN metastasis**	
LN + VE (24/42)	57.14%
LN – VE (18/42)	42.85%

### Ficoll Density Gradient Technique

Peripheral blood mononuclear cells (PBMCs) were isolated using Ficoll (Greiner Bio-One Ltd., Stonehouse, UK), as per the manufacturer's instructions. Harvested cells were washed twice in phosphate buffer saline (PBS, Applied Biosystems; Thermo Fisher Scientific Inc., cat. no. 10010023), and viable cells were counted using a hemocytometer. Cells were frozen in liquid nitrogen at a density of 10^7^ cells/ml in 90% v/v fetal bovine serum (FBS, Applied Biosystems; Thermo Fisher Scientific Inc., cat. no. 10270098) and 10% v/v DMSO (Applied Biosystems; Thermo Fisher Scientific Inc., cat. no. D12345) for later analysis.

### Cell Culture

MDA-MB 231 cells were purchased from Vacsera Egypt. They were incubated in Dulbecco's modified Eagle's medium (DMEM, Lonza, Germany, cat. no. 12-604F) supplemented with 4.5 g/L glucose, 4 mmol/L L-glutamine, 10% FBS (Applied Biosystems; Thermo Fisher Scientific Inc., cat. no. 10270098), and MycoZap (1:500; Lonza, cat. no. LT07-818) at 37°C with an atmosphere of 5% CO_2_ and 95% humidity. The cultured cells were then screened for PD-L1 expression.

### Transfection

Twenty-four hours prior to transfection, seeding of 1–5 × 10^4^ or 2–8 × 10^4^ MDA-MB-231 cells (40–80% confluency) per well of a 96-well plate or 24-well plate, respectively, was performed. The cells were incubated under normal growth conditions (37°C and 5% CO_2_). MDA-MB 231 cells were transfected with siRNAs for both lncRNA XIST (Hs_XIST_3 FlexiTube siRNA, Qiagen Germany, cat. no. SI03654483) and lncRNA TSIX (Hs_TSIX_7 FlexiTube siRNA, Qiagen Germany, cat. no. SI04708795), in addition to performing a negative control using scrambled small interfering RNAs (Scr-siRNAs) that were not targeting lncRNA XIST or TSIX. All transfection experiments were performed in triplicate using HiPerfect Transfection Reagent (Qiagen Germany, cat. no. 301705) according to the manufacturer's instructions, and experiments were repeated three times. Cells that were only exposed to transfection reagent were designated as mock cells, cells transfected with lncRNA XIST siRNA were designated as lncRNA XIST silenced cells, and cells transfected with lncRNA TSIX siRNA were designated as lncRNA TSIX silenced cells. This was followed by RNA extraction, screening for lncRNAs XIST, TSIX, and PD-L1 expression, and finally comparison to MDA-MB 231 mock cells.

### RNA Isolation

RNA was isolated from MDA-MB231 cells, tumor tissues, lymph nodes, PBMCs, serum, and nipple discharge using TRIzol™ LS Reagent (Applied Biosystems; Thermo Fisher Scientific Inc., cat. no. 10296010) extraction protocol.

### Quantified Real-Time Polymerase Chain Reaction (qRT-PCR)

Total RNA extracted was reverse-transcribed into single-stranded cDNA using the high-capacity cDNA reverse transcription kit (Applied Biosystems; Thermo Fisher Scientific Inc., cat. no. 4368814). The relative expression of lncRNAs XIST, TSIX, and PD-L1, along with B actin (as a housekeeping gene for normalization), was quantified and amplified using TaqMan RT-quantitative polymerase chain reaction (qPCR; Applied Biosystems; Thermo Fisher Scientific Inc., Assay IDs: Hs01079824_m1, Hs03299334_s1, Hs00204257_m1, and Hs01060665_g1, respectively) on a StepOne™ Real-Time PCR instrument (Applied Biosystems; Thermo Fisher Scientific Inc.). For every sample, a reaction mix was prepared according to the manufacturer's instructions, and 4 μl of the respective cDNA was added. The RT-qPCR run was performed in the standard mode, consisting of two stages: a first 10-min stage at 95°C where the Taq-polymerase enzyme was activated, followed by a second stage of 40 amplification cycles (15 s at 95 and 60 s at 60°C). Relative expression was calculated using the 2^−ΔΔCT^ method. All PCR reactions including controls were run in triplicate reactions.

### Statistical Analysis

All data were expressed in relative quantitation (RQ). To compare between two different studied groups, Student's unpaired *t*-test was employed. Data were expressed as mean ± standard error of the mean (SEM). A *p* < 0.05 was considered statistically significant. ^****^p < 0.0001, ^***^p < 0.001, ^**^p < 0.01, and ^*^p < 0.05. Analysis was performed using the GraphPad Prism 7.02 software.

## Results

### Expression of PD-L1 in Tumor Tissues and MDA-MB 231 Cells

**Figure 1:**
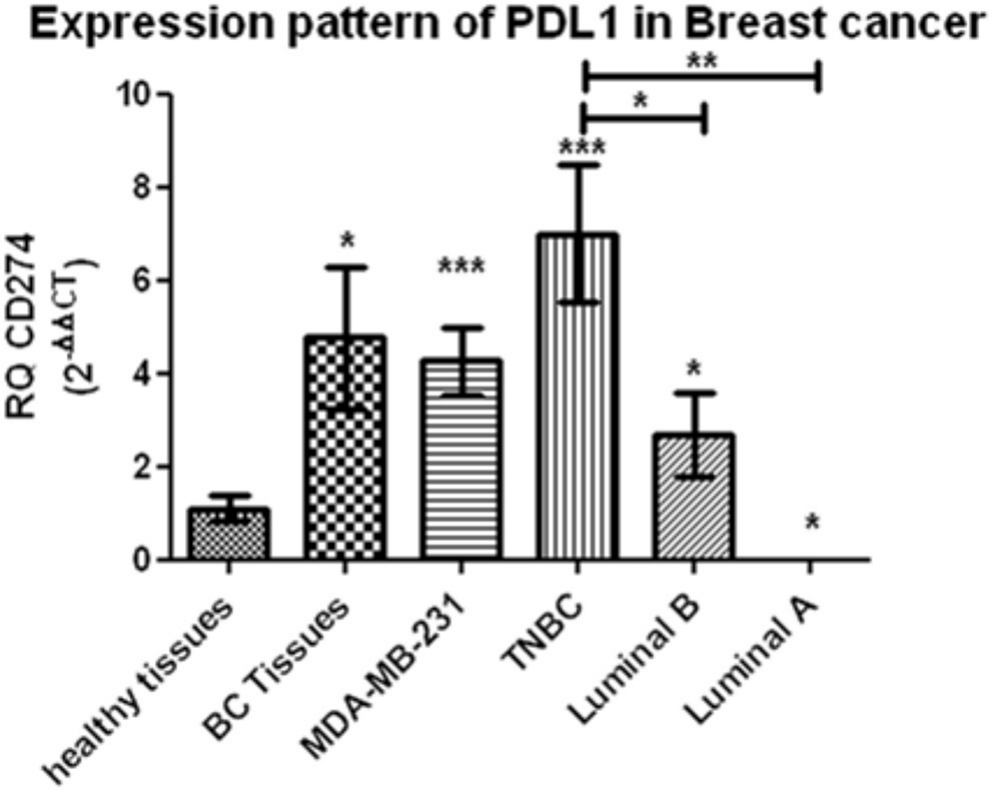
Expression pattern of PD-L1 in breast cancer. A significant upregulation of programmed cell death protein 1 ligand (PD-L1) mRNA has been demonstrated across different breast-cancer tissue subtypes and MDA-MB-231 cells compared to healthy tissues. RQ, relative quantification. ^***^p < 0.001, ^**^p < 0.01, and ^*^p < 0.05.

The entire pool of BC patients as well as MDA-MB 231 cells analyzed displayed a significant upregulation of PD-L1 mRNA (*p* = 0.0101 and *p* = 0.0002, respectively) compared to controls. The results verified the variation in PD-L1 expression across diverse BC patients' molecular subtypes as both luminal B, and TNBC demonstrated significant upregulation of PD-L1 (*p* = 0.0044 and *p* < 0.0001, respectively), in contrast to luminal A who displayed a significant downregulation of PD-L1 (*p* = 0.0181), in addition to a non-significant expression of PD-L1 in HER2/luminal B and HER2/neu compared to controls. Moreover, TNBC exhibited a significant upregulation of PD-L1 compared to luminal B and luminal A samples (*p* = 0.0016 and *p* = 0.0263, respectively) (Figure 1).

### Expression Profiling of lncRNAs XIST and TSIX in Solid Body Sections From Different Molecular Subtypes of BC Patients

**Figure 2:**
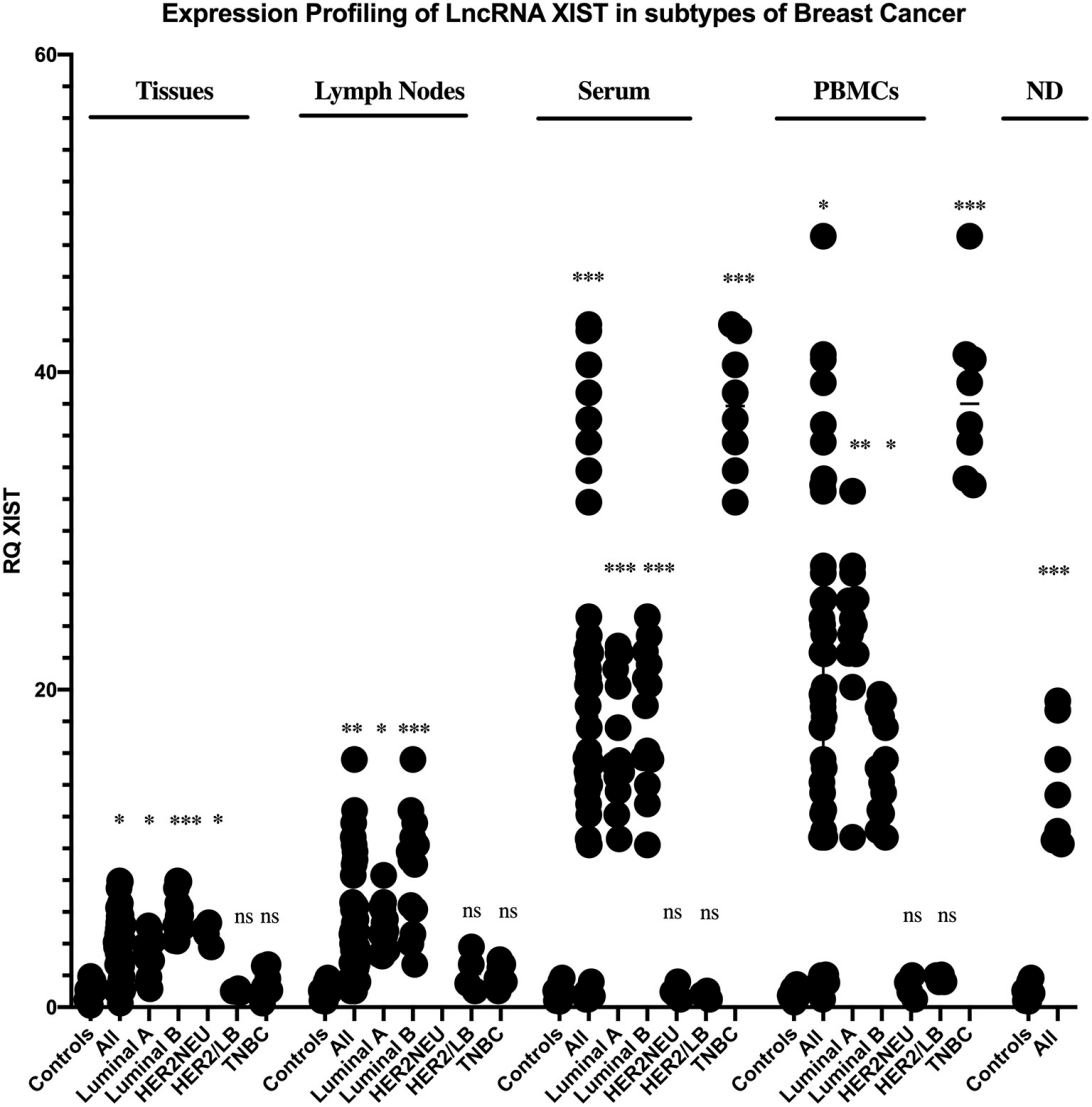
Expression profiling of long non-coding (lnc)RNA XIST in breast cancer (BC) subtypes. Differential expression of lncRNA XIST has been investigated between each BC subtype and control (healthy sample) in addition to its differential expression through different programmed cell death protein 1 ligand (PD-L1)-expressing patients. lncRNA XIST showed a statistically significant downregulation in high-PD-L1-expressing compared to medium-PD-L1-expressing BC tissues and lymph nodes (*p* = 0.047 and *p* = 0.0238, respectively), in contrast to its statistically significant upregulation in high-PD-L1- compared to medium- and low-PD-L1-expressing BC serum and medium-PD-L1-expressing BC peripheral blood mononuclear cells (PBMCs) (*p* = 0.0303, *p* = 0.0003, and *p* = 0.0159, respectively). High-PD-L1 patients denote triple-negative (TN)BCs. Medium PD-L1: luminal B and low PD-L1: luminal A. ND, nipple discharge; RQ, relative quantification. ^***^p < 0.001, ^**^p < 0.01, and ^*^p < 0.05.

**Figure 3:**
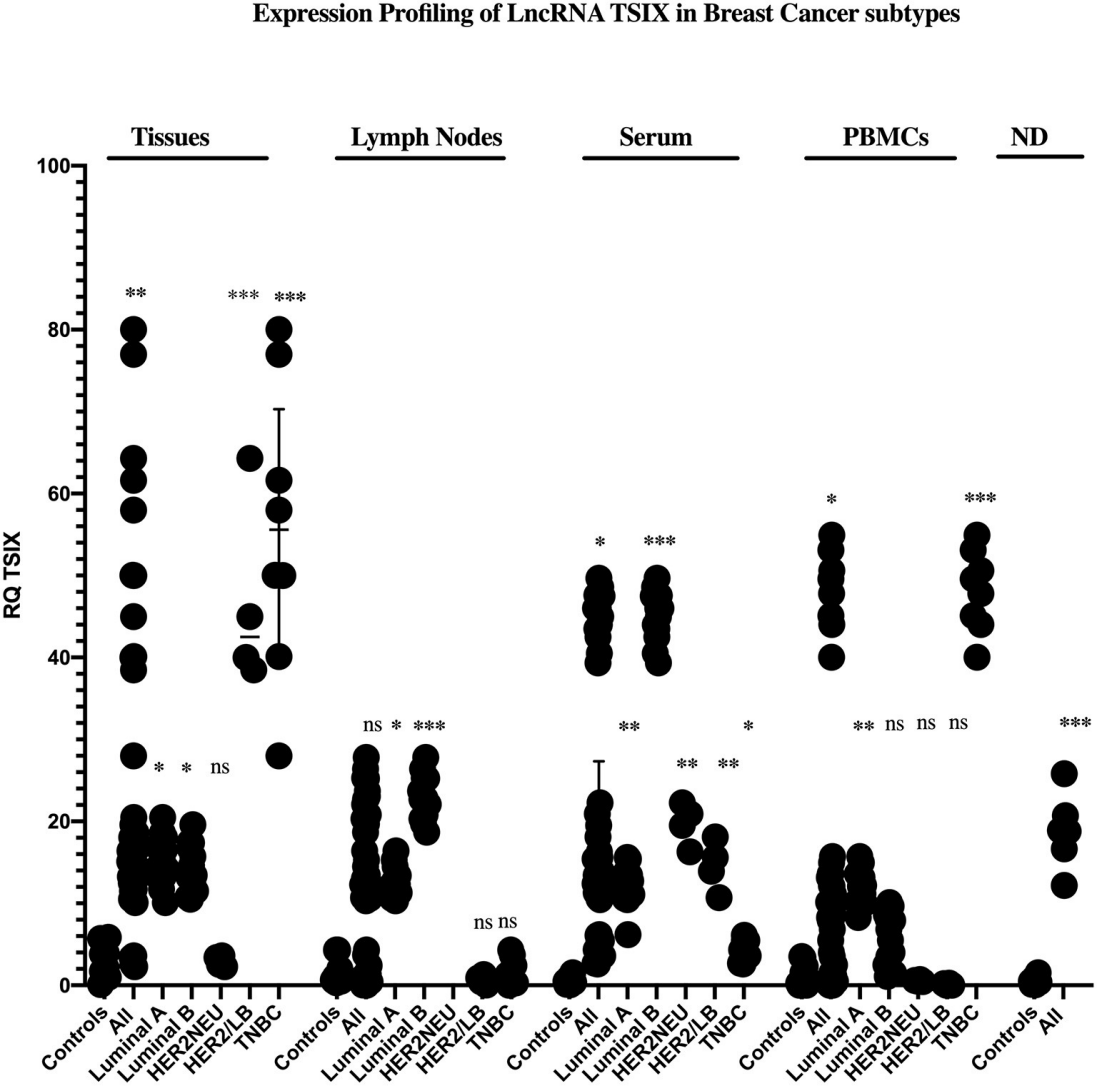
Expression profiling of long non-coding (lnc)RNA TSIX in breast cancer (BC) subtypes. Differential expression of lncRNA TSIX has been analyzed between each BC subtype and control (healthy sample) in addition to its differential expression through different PD-L1-expressing patients. lncRNA TSIX displayed a statistically significant downregulation in high-PD-L1- compared to medium-PD-L1-expressing BC lymph nodes and serum (*p* = 0.0004 and *p* = 0.0047, respectively), in contrast to its statistical significant upregulation in high-PD-L1- compared to medium- and low-PD-L1-expressing BC tissues and medium-PD-L1-expressing BC PBMCs (*p* = 0.0060, *p* = 0.0400, and *p* = 0.0175, respectively). High-PD-L1 patients denote triple-negative (TN)BCs. Medium PD-L1: luminal B and low PD-L1: luminal A. ND, nipple discharge; RQ, relative quantification. ^***^p < 0.001, ^**^p < 0.01, and ^*^p < 0.05.

Starting with BC tissues, there was a statistically significant upregulation in the expression of lncRNAs XIST and TSIX compared to healthy breast tissues (*p* = 0.0353 and *p* = 0.0023, respectively). Upon patient's stratification, both lncRNAs XIST and TSIX were significantly upregulated in luminal A and luminal B subtypes (*p* = 0.0341, *p* < 0.0001, *p* = 0.0400, and *p* = 0.018, respectively) compared to healthy breast tissues (Figures 2, 3, respectively). Furthermore, lncRNA XIST showed significant upregulation in HER2/neu subtype (*p* = 0.0344), although it demonstrated a non-significant change in expression in HER2/luminal B and TNBC subtypes compared to healthy breast tissues (Figure 2), in addition to significant upregulation of lncRNA TSIX in HER2/luminal B and TNBC subtypes (*p* < 0.0001, and *p* < 0.001, respectively), although it demonstrated a non-significant change in expression in HER2/neu subtype compared to healthy breast tissues (Figure 3). To correlate lncRNAs XIST and TSIX expression in tissues to PD-L1 expression, patients were classified into high (TNBC)-, medium (luminal B)-, and low (luminal A)-PD-L1-expressing patients. lncRNA XIST demonstrated significant downregulation in high-PD-L1- compared to medium-PD-L1-expressing patients (*p* = 0.0474). However, there was no significant difference in its expression between high- and low-PD-L1 patients. Additionally, lncRNA TSIX exhibited a significant upregulation in the high- compared to medium- and low-PD-L1-expressing patients (*p* = 0.0060 and *p* = 0.0400, respectively) (Figures 2, 3, respectively).

Proceeding to the lymph nodes, lncRNA XIST displayed a statistically significant upregulation in its expression (*p* = 0.0051), whereas there was a non-significant change in lncRNA TSIX expression in the combined BC patient's lymph nodes compared to non-metastatic lymph nodes. Additionally, lncRNAs XIST and TSIX displayed a significant upregulation in luminal A and luminal B subtypes (*p* = 0.0178, *p* < 0.0001, *p* = 0.0365, and *p* < 0.0001, respectively); nevertheless, they exhibited a non-significant change in expression in HER2/luminal B and TNBC, in addition to absent expression in HER2/neu subtype compared to non-metastatic lymph nodes. Besides, both lncRNAs XIST and TSIX demonstrated a significant downregulation in high compared to medium PD-L1 (*p* = 0.0238 and *p* = 0.0004, respectively), and there was no significant difference in their expression between high- and low-PD-L1 patients (Figures 2, 3, respectively).

### Expression Profiling of lncRNAs XIST and TSIX in Body Fluids From Different Molecular Subtypes of BC Patients

Starting with the serum, both lncRNAs XIST and TSIX displayed a statistically significant upregulation (*p* = 0.0007 and *p* = 0.0363, respectively) compared to controls. Additionally, they demonstrated a significant upregulation in luminal A, luminal B, and TNBC subtypes (*p* = 0.0003, *p* = 0.0003, *p* < 0.0001, *p* = 0.0016, *p* < 0.0001, and *p* = 0.0315, respectively) compared to controls (Figures 2, 3, respectively). Furthermore, lncRNA XIST exhibited a non-significant change in expression in HER2/luminal B and HER2/neu subtypes in contrast to lncRNA TSIX, which showed a significant upregulation in both subtypes compared to controls (*p* = 0.0012 and *p* = 0.0099, respectively). Moreover, lncRNA XIST displayed significant upregulation in high-PD-L1 compared to patients expressing medium and low PD-L1 (*p* = 0.0303 and *p* = 0.0003, respectively). As for lncRNA TSIX, it presented significant downregulation in high- compared to medium-PD-L1-expressing patients (*p* = 0.0047), yet there was no significant difference in its expression between high and low PD-L1 (Figures 2, 3, respectively).

There was a significant upregulation in the expression of lncRNAs XIST and TSIX in PBMCs of BC patients compared to controls (*p* = 0.0499 and *p* = 0.0242, respectively). Additionally, they displayed a significant upregulation in luminal A and TNBC subtypes (*p* = 0.0063, *p* < 0.0001, *p* = 0.0026, and *p* < 0.0001, respectively), in addition to a non-significant change in their expression in HER2/luminal B and HER2/neu subtypes compared to controls. Furthermore, lncRNA XIST showed a statistical significant upregulation in luminal B subtype (*p* = 0.00343) in contrast to TSIX lncRNA, which exhibited a non-significant change in expression in luminal B compared to controls. Finally, both lncRNAs exhibited significant upregulation in high- compared to medium-PD-L1-expressing patients (*p* = 0.0159 and *p* = 0.0175, respectively). However, there was no significant difference in their expression between high- and low-PD-L1 patients (Figures 2, 3, respectively).

Finally, as for the nipple discharge, there was a statistically significant upregulation of both lncRNAs XIST and TSIX compared to controls (*p* = 0.0008 and *p* < 0.0001, respectively). Additionally, they displayed a significant upregulation in both luminal B and TNBC subtypes compared to controls (*p* < 0.0001, *p* = 0.0093, *p* = 0.0008, and *p* < 0.0001, respectively). Furthermore, lncRNA XIST displayed significant downregulation in high- compared to medium-PD-L1-expressing patients (*p* = 0.0284), in contrast to lncRNA TSIX, which showed a non-significant difference between high- and medium-PD-L1 patients (Figures 2, 3, respectively). Collectively, all these results evidenced that the pattern of expression of both lncRNAs XIST and TSIX in the solid body sections can be reflected in the different non-invasive body fluids of BC patients, thus confirming the role of XIST and TSIX lncRNAs as non-invasive immune biomarkers in BC patients.

### Expression of lncRNAs XIST and TSIX in Pre- and Post- menopausal BC Patients

**Figure 4:**
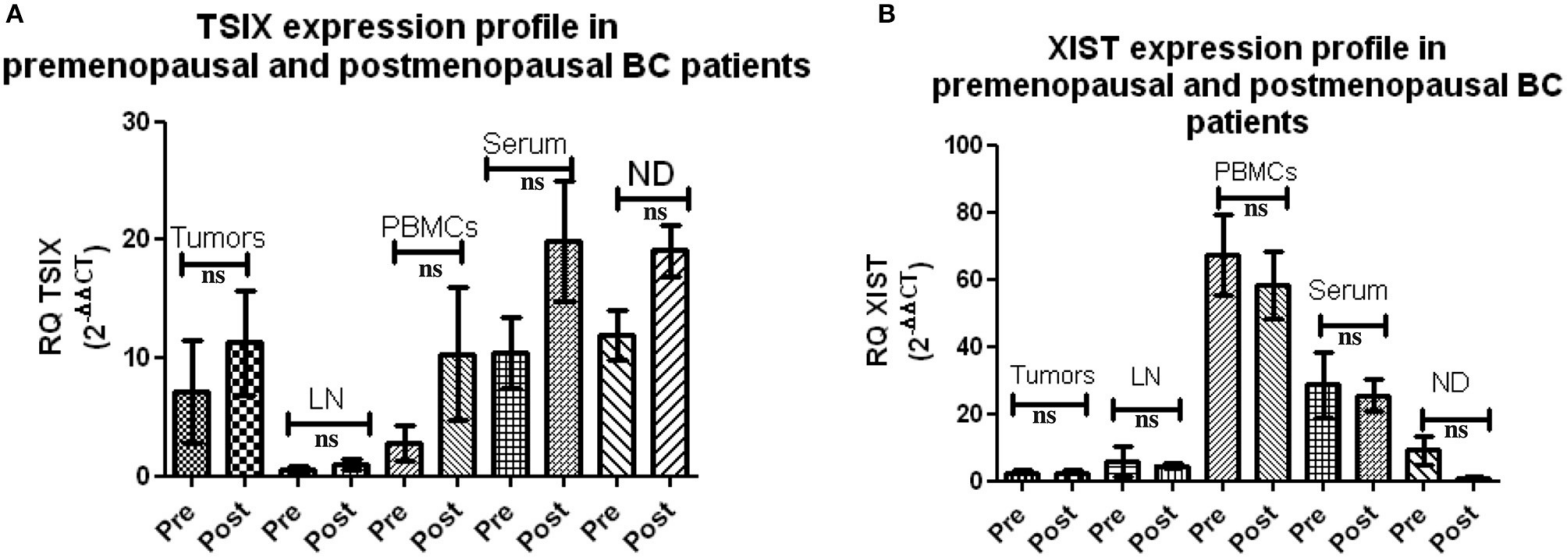
Expression of long non-coding (lnc)RNAs XIST and TSIX in pre- and postmenopausal breast cancer (BC) patients. **(A,B)** Screening using qRT-PCR revealed a non-significant difference in the pattern of expression of both lncRNAs XIST and TSIX in solid body sections and different body fluids of pre- compared to postmenopausal BC patients, respectively. ND, nipple discharge; RQ, relative quantification; ns, non-significant.

Extending the aim of this study to unraveling hormonal impact on both lncNRAs XIST and TSIX expression, BC patients were divided into pre- and postmenopausal groups. Both lncRNAs XIST and TSIX displayed non-significant differences in their expression in the tissues, lymph nodes, and different body fluids of premenopausal BC patients compared to the postmenopausal BC patients (Figure 4), respectively, demonstrating that their level is unaffected by hormonal changes.

### Impact of lncRNAs XIST and TSIX Knockdown on PD-L1 mRNA Expression Level in MDA-MB-231 Cells

**Figure 5:**
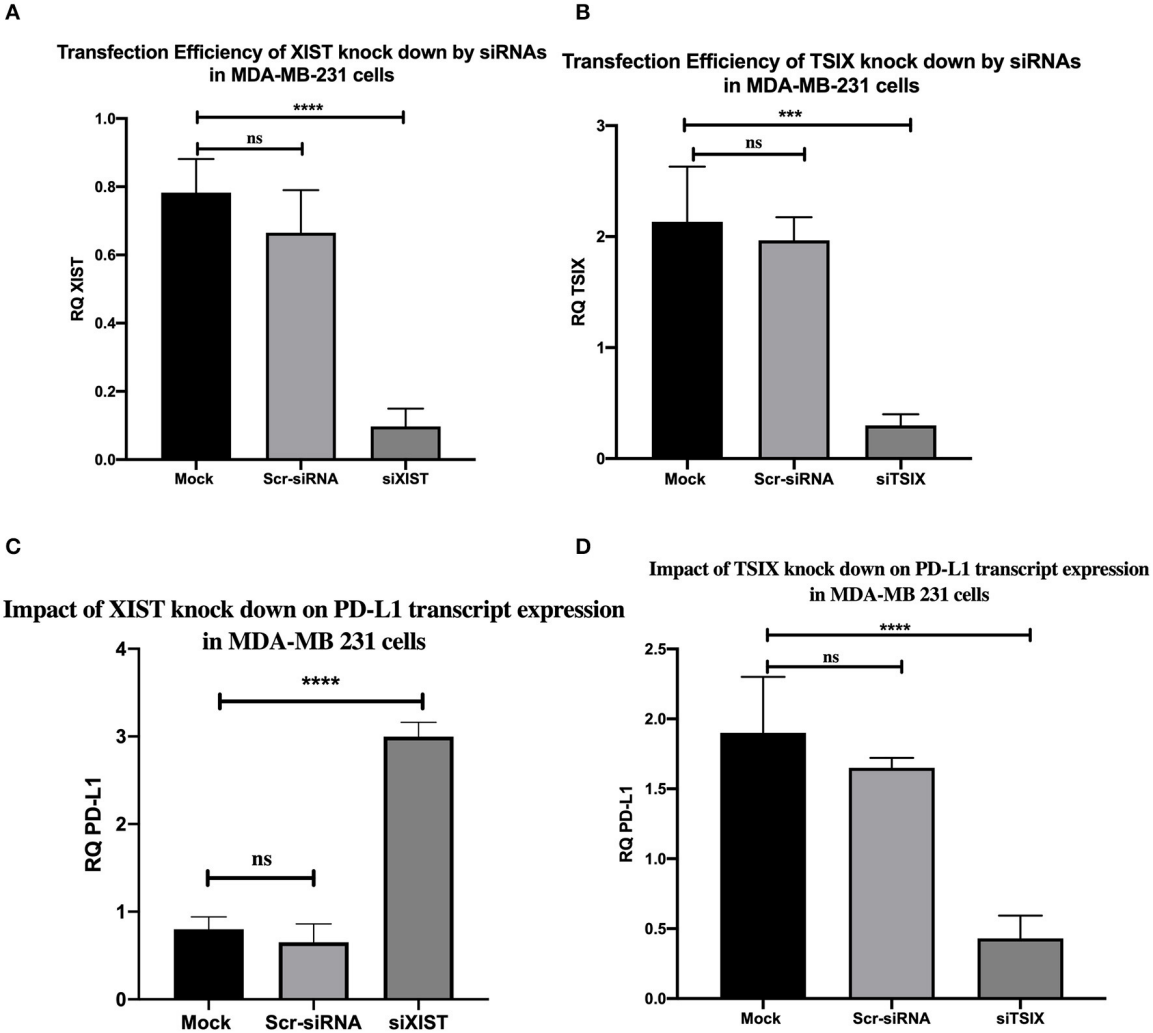
Transfection efficiency of long non-coding (lnc)RNAs XIST and TSIX knockdown by siRNAs and the impact of their knockdown on programmed cell death protein 1 ligand (PD-L1) mRNA expression in MDA-MB-231 cells. **(A,B)** Confirmed efficient knockdown of lncRNAs XIST and TSIX compared to mock cells. **(C,D)** Knockdown impact of lncRNAs XIST and TSIX on PD-L1 expression. RQ, relative quantification. ^****^p < 0.0001; ^***^p < 0.001.

Finally, to give weight to the correlation between lncRNAs XIST, TSIX, and PD-L1 expressions, the impact of lncRNAs XIST and TSIX in PD-L1 expression was investigated through their knockdown. Efficient knockdown of both lncRNAs XIST and TSIX in MDA-MB231 cells was confirmed (*p* = 0.0230 and *p* = 0.0387, respectively). As a consequence to lncRNA XIST repression, a dramatic elevation of PD-L1 was observed (*p* < 0.0001). However, a remarkable repression of PD-L1 was witnessed upon lncRNA TSIX repression (*p* = 0.0005) (Figure 5). There existed a non-significant difference between the mock and scrambled siRNA controls. For these data, an inverse relationship between XIST and PD-L1 has been observed, in contrast to a direct relationship between TSIX and PD-L1 expression.

## Discussion

**Figure 6:**
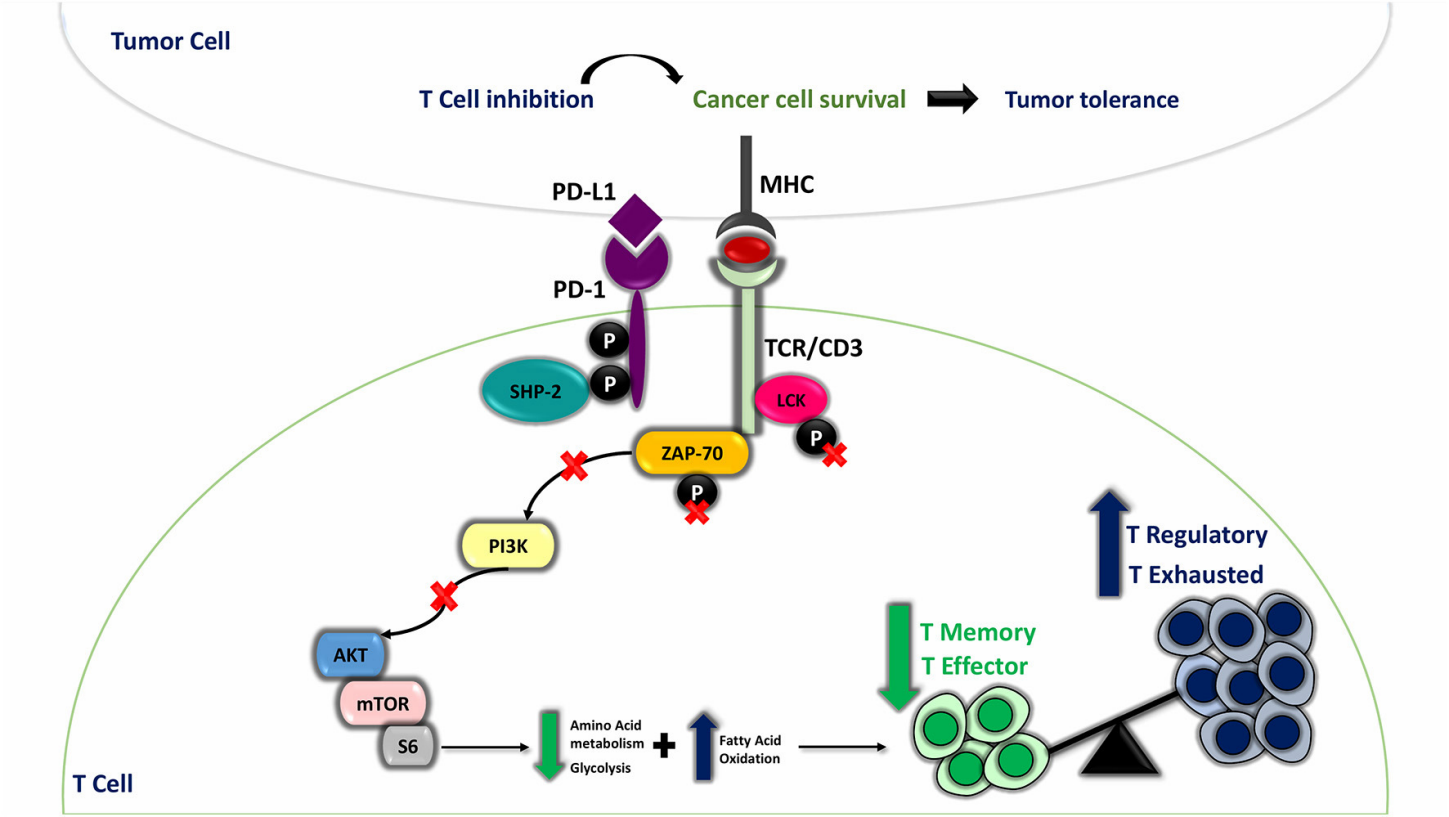
Programmed cell death protein 1 (PD-1) ligand (PD-L1) involvement in T-cell mediated suppression and maintenance of immune evasion. Under physiological conditions, T cell is activated against any foreign antigen presented on the surface of antigen-presenting cell through major histocompatibility complex (MHC) by means of T-cell receptor (TCR) phosphorylation when oligomerization of TCR/CD3 chains takes place; this is followed by recruitment of activated Lck and Zap-70 to the phosphorylated immunoreceptor tyrosine-based activation motif (ITAM) of TCR tail, and this leads to the start of downstream TCR-signaling cascade. In cancer state, when PD-L1 present on cancer cells interacts with PD-1 expressed on T-cell surface, SHP-2 is recruited to ITSM, and the two tyrosine residues on PD-1 cytoplasmic tail acquire a phosphorylated status. This will result in dephosphorylated Lck and Zap-70 and hence their inactivation. Furthermore, PD-L1-PD-1 ligation suppresses PI3K/AKT/mTOR pathway, which affects normal T-cell metabolic pathway, shifting the pathway toward fatty acid oxidation (FAO) instead of glycolysis and amino acid metabolism, resulting in creation of immune suppressive milieu through enhanced T regulatory cells (immune suppressive cells) and exhausted T-cell differentiation and impaired effector and memory T cells differentiation.

While PD-1/PD-L1 pathway is pivotal to sustain an equilibrium between autoimmunity and peripheral tolerance, it impairs tumor immunity when employed by cancer cells promoting immune suppression and immune surveillance escape (34). For instance, PD-L1 overexpression in TNBC subtype establishes an immunosuppressive milieu through T-cell metabolic program alteration, leading to impaired effector and memory T-cell differentiation and prevalence of immunosuppressive T cells (regulatory T cells) and exhausted T cells in peripheral blood of TNBC patients (Figure 6) (34, 35). Owing to the advances in the implementation of PD-L1 in BC immunotherapy (14, 15), the emergence of novel immune biomarkers that can surpass the limitations of current immune biomarkers is highly warranted (22, 23). lncRNAs represent ideal candidates as novel BC immune biomarkers due to their stability and their massive involvement in BC carcinogenesis (24, 25, 36–38). A mounting body of evidence highlighted different contributing roles of the lncRNA XIST either as an oncogene or as a tumor suppressor gene in diverse cancers (39–43). Nonetheless, limited evidence displayed lncRNA XIST involvement as a tumor suppressor lncRNA in BC (28, 29) in addition to the absence of concerns regarding lncRNA TSIX, the negative regulator of lncRNA XIST in BC. Furthermore, the correlation between XIST, TSIX, and PD-L1 so far is largely unexplored. This study was mainly focused on revealing the role of lncRNAs XIST and TSIX as potential stable non-invasive BC immune biomarkers in different liquid body fluids and correlating their expression to PD-L1 expression.

Thus, it was essential, first, to compare PD-L1 expression in different BC patient subtypes and TNBC cells to controls. TNBCs were found to have a significant high PD-L1 expression followed by luminal B and luminal A patients (Figure 1). And this is in accordance with previous studies that have put forward evidence that PD-L1 was significantly overexpressed in BC patients (44, 45). TNBC subtype will be referred to as high-PD-L1-expressing cells and luminal A and luminal B subtypes will be referred to as low-PD-L1-expressing cells.

Both lncRNAs XIST and TSIX expressions in tissues and for the first time lymph nodes of BC patients were compared to controls; our results displayed the significant upregulation of both lncRNAs XIST and TSIX in the BC patient tissues compared to healthy breast tissues. lncRNA XIST also displayed significant upregulation in malignant lymph nodes compared to benign lymph nodes (Figures 2, 3, respectively).

In harmony with our results, lncRNA XIST was proven to be upregulated in different types of cancer tissues compared to healthy tissues. For instance, the overexpressed lncRNA XIST in colorectal cancer promoted cell proliferation and invasiveness through controlling ZEB1 expression by acting as a ceRNA for miR-200b-3p (40). Upregulated lncRNA XIST in hepatocellular carcinoma was found, in another study, to activate AKT pathway through positive regulation of PDK1 expression *via* inhibiting miR-139-5p (41). However, other studies were not in line with our results and have proven the downregulation of lncRNA XIST in metastatic BC tissues. The downregulated lncRNA XIST enhanced BC tissue growth, migration, and invasiveness; upon upregulating lncRNA XIST BC tissues, migration and invasive capability were inhibited through miR-155/CDX1 axis (29). Another study reported that both lncRNAs XIST and TSIX were downregulated in BC tissues.

**Figure 7:**
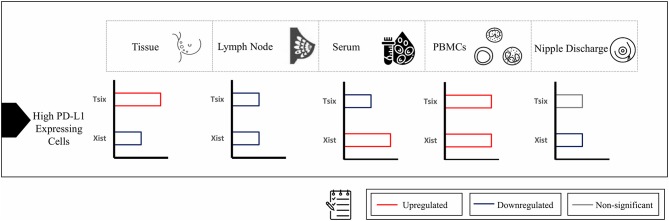
Summary of expression of long non-coding (lnc)RNAs XIST and TSIX in high-programmed cell death protein 1 ligand (PD-L1)-expressing cells compared to low-PD-L1-expressing cells in different body parts. In high-PD-L1-expressing cells of breast-cancer tissues, there was significant downregulation of lncRNA XIST and upregulation of lncRNA TSIX compared to low-PD-L1-expressing cells; however, in lymph nodes, high-PD-L1-expressing cells showed significant downregulation of both lncRNAs XIST and TSIX compared to low-PD-L1-expressing cells. As for different body fluids investigated, the serum high-PD-L1-expressing cells displayed significant upregulation of lncRNA XIST and downregulation of lncRNA TSIX compared to low-PD-L1-expressing cells. In peripheral blood mononuclear cells (PBMCs), high-PD-L1-expressing cells demonstrated significant upregulation of both lncRNAs XIST and TSIX compared to low-PD-L1-expressing cells. Finally, in nipple discharge, high-PD-L1-expressing cells exhibited significant downregulation of lncRNA XIST compared to low-PD-L1-expressing cells.

Downregulating lncRNA XIST in BC tissues improved the viability of BC cells through repressed expression of PHLPP1 (a phosphatase-inhibiting AKT phosphorylation) *via* increased HDAC3 recruitment to the PHLPP1 promoter, which had a mediatory effect on upregulation of AKT phosphorylation with no mention of any role for the downregulated TSIX (28). Additionally, upregulated lncRNA XIST in malignant lymph nodes was in alliance with further studies, postulating the candidate biomarker role of upregulated CD-169 (46), NUCB2 (47), and P-cadherin (48) in BC patients malignant lymph nodes. Furthermore, upon stratifying patients based on PD-L1 expression, high-PD-L1-expressing cells in BC tissues showed significant downregulation of lncRNA XIST and upregulation of lncRNA TSIX compared to low-PD-L1-expressing cells. In lymph nodes, high-PD-L1-expressing cells showed significant downregulation of both lncRNAs XIST and TSIX compared to low-PD-L1-expressing cells (Figure 7).

As serum, nipple discharge, and PBMCs represent ideal non-invasive samples, it was mandatory to investigate whether they are capable of mirroring tissue and lymph node lncRNAs XIST and TSIX expression pattern and hence proving the lncRNAs XIST and TSIX role as potential stable non-invasive BC immune biomarkers. In this study, our data exhibited that both lncRNAs XIST and TSIX were significantly upregulated in the whole pool of BC serum, PBMCs, and nipple discharge compared to controls (Figures 2, 3, respectively). These results go in line with previous studies highlighting upregulated lncRNA XIST in non-small-cell lung cancer (49), lncRNA MALAT-1, lncRNA HOTAIR (50), miR-155 (51), and IL-6 (52) as upregulated serum BC patient diagnostic biomarkers. Moreover, our findings also go in line with previous studies that highlighted lncRNA XIST as an upregulated diagnostic biomarker in PBMCs of HCC patients (53) and miR-4484, miRK12-5-5p, and miR-3646 as upregulated biomarkers in nipple discharge of BC patients (54). In serum, high-PD-L1-expressing cells revealed significant upregulation of lncRNA XIST and downregulation of lncRNA TSIX compared to low-PD-L1-expressing cells, while in PBMCs, high-PD-L1-expressing cells showed significant upregulation of both lncRNAs XIST and TSIX compared to low-PD-L1-expressing cells, and finally, in nipple discharge, high-PD-L1-expressing cells demonstrated significant downregulation of lncRNA XIST compared to low-PD-L1-expressing cells (Figure 7).

To account for the discrepancy in lncRNA XIST expression in different body fluids, our hypothesis was that—based on the fact that ncRNAs expressed in the tissues can be released in a variety of body fluids (55, 56)—the low lncRNA XIST expression in high-PD-L1-expressing nipple discharge cells is a result of its major release from tissues to serum rather than nipple discharge. An explanation for the high level of lncRNA XIST in high-expressing-PD-L1 PBMC cells can be explained by the fact that PBMCs have their own RNA machinery (57), which enables them to synthesize lncRNA XIST in the form of a counterregulatory response to combat immune evasion resulting from PD-L1 overexpression.

To prove XIST and TSIX lncRNAs hormonal independency, BC patients were subdivided into pre- and postmenopausal patients. The data reflected no significant difference between pre- and postmenopausal women in terms of lncRNAs XIST and TSIX expression in different BC patient's body sections investigated (Figure 4). This is countered by other studies holding that some biomarkers are affected by hormonal changes such as serum level of vitamin D, which was stated to be lowly expressed in perimenopausal women (58) and lncRNA HOTAIR, which was demonstrated to be dependent on estradiol level (59). Moreover, it should be noted that the postulation of pregnancy-induced protective effect against BC was not supported in our pool of patients as 85 and 15% of female patients were multiparous and nulliparous, respectively, in addition to two male patients, where all of the three groups had aggressive phenotypes of BC in our study. This is in opposition to a study indicating that early pregnancy reduces the risk of postmenopausal BC development in women through induction of chromatin remodeling in the breast of parous women compared to nulliparous women. This remodeling resides in a shift of nuclear state and appearance of cells forming lobules type 1 (lob 1) in parous breast to small and hyperchromatic nuclei with strong gene methylation and silencing, which is thought to be initiated by upregulated lncRNA XIST in parous breast compared to nulliparous women (60).

Finally, digging deeper to explore a correlation between lncRNAs XIST, TSIX, and PD-L1 expression in BC patients, TNBC cells (MDA-MB231 cells) were transfected with siRNAs for both lncRNAs XIST and TSIX followed by monitoring their knockdown impact on PD-L1 expression. The results disclosed a reverse association between PD-L1 and lncRNA XIST, where, upon lncRNA XIST knockdown, there was significant induction in PD-L1 expression, in contrast to lncRNA TSIX that significantly decreased PD-L1 expression upon its knockdown (Figure 5). This was consistent with previous reports in which lncRNA XIST was shown to act as a tumor suppressor in BC (28, 29) and with the fact that lncRNA TSIX is a negative regulator of lncRNA XIST (61).

**Figure 8:**
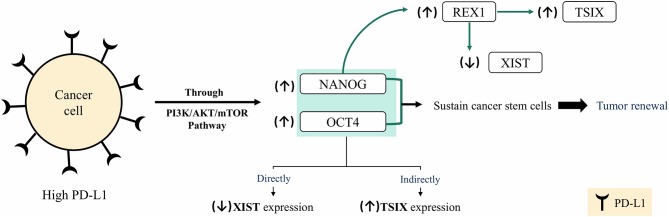
Hypothesis of XIST/TSIX/PD-L1 regulation. In cancer cells, high- programmed cell death protein 1 ligand (PD-L1) expression sustains high level of both OCT4 and NANOG *via* PI3K/AKT/mTOR pathway activation, which results in sustained cancer cell stemness and tumor renewal. High level of OCT4 and Nanog will affect XIST and TSIX expression, resulting in repression of XIST expression and maintenance of high TSIX expression.

To draw the full hypothesis regarding the mechanism by which XIST/TSIX/PD-L1 interacts, literature was screened. Based on the literature review, it was evident that the recruitment of NANOG, OCT4, and REX1 has been implicated in the regulation of lncRNAs XIST and TSIX expression. One of the suggested mechanisms by which pluripotency factors NANOG and OCT4 directly repress lncRNA XIST expression is through binding to a region present within lncRNA XIST intron 1 (62), which was proven later to be dispensable for X chromosome inactivation (63), because deletion of intron 1 only mildly affects XCI as pluripotency factors act in concert toward inhibiting expression of XIST *via* binding to different sites present throughout the XIST (64) in addition to the indirect activation of lncRNA TSIX expression (64). REX1 recruitment is involved in direct repression of lncRNA XIST (65) as well as lncRNA TSIX activation and upregulation through binding to a regulatory region present in mice (called DPXas34) in lncRNA TSIX (66). Furthermore, NANOG positively regulates REX1 expression through binding to pluripotency-related transcription factor binding site located on REX1 promoter (67). Altogether, NANOG and OCT4 expression was correlated to PD-L1 expression and to the maintenance of BC stemness as highly expressed PD-L1 maintained expression of OCT4 and NANOG through PI3K/AKT/mTOR pathway activation in TNBC cells (68). Accordingly, XIST/TSIX/PD-L1 interaction resides in the high expression of PD-L1, which maintains NANOG and OCT4 expression; this will straightforwardly exert a repressing effect on lncRNA XIST expression and indirectly overexpresses lncRNA TSIX expression (Figure 8), yet this hypothesized interaction merits more detailed studies to ascertain its validity.

In addition to the uncertain mechanism of interaction between lncRNAs XIST, TSIX, and PD-L1, another limitation of this study is the unrevealed mechanistic contribution of both lncRNAs XIST and TSIX in BC progress as our main concern was exploring a correlation between lncRNAs XIST, TSIX, and PD-L1 expression in BC patients. Nevertheless, this study is strengthened by providing the first evidence of expression of both lncRNAs XIST and TSIX in lymph nodes, serum, PBMCs, and nipple discharge of BC patients correlated to PD-L1 expression in addition to excluding their hormonal dependency and finally in shedding the light upon their significant non-invasive immune biomarker role that reflects the immune checkpoint (PD-L1) expression status in BC cells, which can be further utilized in the field of personalized medicine to ascertain—with no invasive means—the eligibility of BC patients for anti-PD-L1 treatment.

In conclusion, this study crystallizes the role of lncRNAs XIST and TSIX as stable non-invasive immune biomarkers better tolerated by patients compared to conventional biopsies with a potential role in reflecting the evaded immune status of BC patients.

## Data Availability Statement

All datasets generated for this study are included in the article/Supplementary Material.

## Ethics Statement

The studies involving human participants were reviewed and approved by German University in Cairo and Ain Shams University Ethical Committees. The patients/participants provided their written informed consent to participate in this study.

## Author Contributions

ES has performed all the lab work and wrote the manuscript. RA is the clinical oncologist and surgeon who was responsible for providing all samples and clinical data. HE is the principal investigator and the main supervisor of this research work and has read and approved the final manuscript.

### Conflict of Interest

The authors declare that the research was conducted in the absence of any commercial or financial relationships that could be construed as a potential conflict of interest.
