# Supplementary material for: XIST and TSIX: Novel Cancer Immune Biomarkers in PD-L1-Overexpressing Breast Cancer Patients
Source: Front Oncol. 2020 Jan 10;9:1459. doi: 10.3389/fonc.2019.01459 (PMC6966712; doi:10.3389/fonc.2019.01459)
Supplement: Supplementary file 1 [file Table_1.DOCX]

**Table1 : Breast Cancer patients Clinical features**

| Patient | Age | Size of mass cm | Type | Grade | Stage | Ki67 | CA 15.3 | Duration | Molecular  Subtype | Axillary  Lymph  Node | Treatment |
| --- | --- | --- | --- | --- | --- | --- | --- | --- | --- | --- | --- |
| Patient 1 | 62 | 4cm | IDC | 3 | 4 | 35% | 73.7 | 2 months | Triple -ve | +ve | N/A |
| Patient 2 | 40 | 2 cm | IDC | 2 | 2 | 35% | 70 | 1 months | Triple -ve | +ve | N/A |
| Patient 3 | 56 | 3cm | IDC | 2 | 2 | 85% | 10 | 4 months | Triple -ve | -ve | N/A |
| Patient 4 | 62 | 2.5cm | IDC | 2 | 2 | 50% | 10 | 3 months | Luminal B | +VE | N/A |
| Patient 5 | 38 | 0.3cm | IDC | 2 | 1 | 30% | 42 | 1 month | HER 2 +VE | +VE | N/A |
| Patient 6 | 74 | 3 cm | IDC | 2 | 2 | 20% | 10 | 4 months | Lum A | +ve | N/A |
| Patient 7 | 69 | 4 cm | ILC | 2 | 2 | 18% | 8.7 | 6 months | Lum A | +VE | N/A |
| Patient 8 | 56 | 1.5cm | IDC | 1 | 1 | 14 | 16 | 2 years | Lum A | _VE | N/A |
| Patient 9 | 28 | 6 cm | IDC | 2 | 2 | 5% | 34.2 | 6 months | Lum A | +VE | N/A |
| Patient 10 | 54 | 2.5cm | IDC | 3 | 3 | 18% | 8.5 | 10 months | Triple -VE | +VE | N/A |
| Patient 11 | 45 | 2.5 cm | IDC | 2 | 2 | 10% | 26.4 | 2 years | Lum A | -VE | N/A |
| Patient 12 | 44 | 2.5 cm | IDC | 2 | 2 | 35% | 48.2 | 7 months | Lum B | +VE | N/A |
| Patient 13 | 67 | 2.5cm | IDC | 2 | 3 | 22% | 15 | 8 months | Lum B | +VE | Neoadjuvant chemotherapy |
| Patient 14 | 56 | 4 cm | IDC | 3 | 4 | 60% | 48.7 | 6 months | Lum B | -VE | Neoadjuvant chemotherapy |
| Patient 15 | 59 | 1 cm | IDC | 3 | 2 | 35% | 36.2 | 6 months | LumB HER2NEU | +VE | N/A |
| Patient 16 | 42 | 9 cm | IDC | 2 | 2 | 50% | 18 | 1 year | Lum B | -ve | N/A |
| Patient 17 | 59 | 4.2 cm | IDC | 2 | 2 | 30% | 18.7 | 1 months | Lum B | +ve | N/A |
| Patient 18 | 43 | 1.6cm | ILC | 1 | 2 | 22% | 13 | 2 months | Lum B | -ve | N/A |
| Patient 19 | 58 | 2.5 cm | IDC | 2 | 2 | 35% | 38 | 6 months | HER2 +VE | +VE | N/A |
| Patient 20 | 45 | 1.5cm | IDC | 2 | 1 | 8% | 32.5 | 4 months | Lum A | +VE | N/A |
| Patient 21 | 64 | 3.5 cm | IDC | 2 | 2 | 30% | 35.8 | 4 months | Triple -ve | +VE | N/A |
| Patient 22 | 31 | 1.8cm | IDC | 2 | 1 | 7% | 18 | 1 months | Lum A | -ve | N/A |
| Patient 23 | 32 | 2 cm | IDC | 2 | 2 | 30% | 13 | 4 months | Triple -VE | +ve | N/A |
| Patient 24 | 54 | 2.5 cm | IDC | 2 | 2 | 18% | 12 | 2 months | Lum A | +ve | N/A |
| Patient 25 | 70 | 4cm | IDC | 2 | 4 | 15% | 40 | 6 months | Lum A | +VE | N/A |
| Patient 26 | 63 | 4 cm | IDC | 3 | 1 | 40% | 26.2 | 2 months | Luminal B | +VE | N/A |
| Patient 27 | 54 | 2.4 cm | IDC | 2 | 2 | 40% | 31.5 | 6 months | Luminal B her2 neu | +ve | N/A |
| Patient 28 | 57 | 4 cm | IDC | 3 | 2 | 15% | 17 | 6 months | Luminal B | +VE | N/A |
| Patient 29 | 53 | 2.5 cm | IDC | 2 | 3 | 25% | 6.6 | 8 months | Luminal B her 2 neu | -Ve | Neoadjuvant chemotherapy |
| Patient 30 | 70 | 2.5 cm | IDC | 2 | 2 | 23% | 18 | 2 months | Luminal B | +VE | N/A |
| Patient 31 | 55 | 14 mm | IDC | 2 | 1 | 12% | 11.4 | 2 months | Luminal A | -VE | N/A |
| Patient 32 | 61 | 2.1cm | IDC | 2 | 2 | 23% | 29 | 4 months | Luminal B | -vE | N/A |
| Patient 33 | 45 | 1.4 cm | IDC | 2 | 1 | 30% | 48 | 3 months | Luminal B | -VE | N/A |
| Patient 34 | 65 | 3.5 cm | IDC | 2 | 2 | 17% | 10 | One year | Luminal A | -VE | N/A |
| Patient 35 | 40 | 5 cm | IDC | 3 | 3 | 50% | 70 | 6 months | HER2 neu | -VE | N/A |
| Patient 36 | 40 | 0.3 cm | IDC | 2 | 2 | 30% | 11.1 | 1 months | TNBC | -VE | chemotherapy |
| Patient 37 | 60 | 2 cm | IDC | 2 | 2 | 35% | 40 | 1 months | TNBC | -VE | N/A |
| Patient 38 | 59 | 2.7 cm | IDC | 2 | 2 | 30% | 18 | 2 weeks | Luminal B HER2 neu | +ve | N/A |
| Patient 39 | 65 | 4.2 cm | IDC | 2 | 2 | 40% | 37.3 | 6 months | HER2 neu | -ve | N/A |
| Patient 40 | 45 | 2 cm | IDC | 2 | 2 | 14% | 28.2 | 2 months | Luminal A | -VE | N/A |
| Patient 41 | 62 | 2 cm | IDC | 2 | 2 | 18% | 10 | 1 months | Luminal A | -ve | Chemo &radio  therapy |
| Patient 42 | 40 | 4 cm | IDC | 2 | 2 | 24% | 14.3 | 8 months | Luminal B | +VE | N/A |
